# Zika virus infection during the period of maximal brain growth causes microcephaly and corticospinal neuron apoptosis in wild type mice

**DOI:** 10.1038/srep34793

**Published:** 2016-10-07

**Authors:** Wen-Chin Huang, Rachy Abraham, Byoung-Shik Shim, Hyeryun Choe, Damon T. Page

**Affiliations:** 1 Department of Neuroscience, The Scripps Research Institute, Jupiter, Florida, USA; 2 The Doctoral Program in Chemical and Biological Sciences at The Scripps Research Institute, Jupiter, Florida, USA; 3 Department of Immunology and Microbial Science, The Scripps Research Institute, Jupiter, Florida, USA.

## Abstract

Zika virus (ZIKV) infection in pregnant women has been established as a cause of microcephaly in newborns. Here we test the hypothesis that neurodevelopmental stages when the brain is undergoing rapid growth are particularly vulnerable to the effects of ZIKV infection. We injected ZIKV intracranially into wild type C57BL/6 mice at two different time points: early postnatal development, when the brain is growing at its maximal rate, and at weaning, when the brain has largely reached adult size. Both time points showed widespread immunoreactivity for ZIKV and cleaved caspase 3 (CC3, a marker of apoptosis) throughout the brain. However, in early postnatal ZIKV injected mice, some brain areas and cell types display particularly large increases in apoptosis that we did not observe in older animals. Corticospinal pyramidal neurons, a cell type implicated in human microcephaly associated with ZIKV infection, are an example of one such cell type. Proliferating cells in the ventricular zone stem cell compartment are also depleted. These findings are consistent with the hypothesis that periods of rapid brain growth are especially susceptible to neurodevelopmental effects of ZIKV infection, and establish a valuable model to investigate mechanisms underlying neurodevelopmental effects of ZIKV infection and explore candidate therapeutics.

Zika virus (ZIKV) is a flavivirus that was first isolated in rhesus macaque in Uganda in 1947. Transmitted by mosquito, it is related to several other human pathogens, including West Nile virus, dengue, Japanese encephalitis virus and yellow fever. A ZIKV epidemic in Brazil in 2015 coincided with a dramatic increase in cases of microcephalic newborns. Several lines of evidence from human cases and animal studies indicate an association between ZIKV infection during pregnancy and abnormal fetal development^1^,^2^,^3^,^4^,^5^,^6^,^7^ A prospective study of ZIKV-infected pregnant women has reported abnormalities, including microcephaly and intrauterine growth restriction, in 29% of fetuses^2^. Initial reports indicate that all trimesters of pregnancy are vulnerable to the effects of maternal ZIKV infection on fetal development, with the most severe phenotypes associated with infection during the first or second trimester^2^. Although information about the neuropathological characteristics of microcephaly caused by ZIKV infection is extremely limited at this time, a case report of a fetus aborted in the 32^nd^ gestational week has recently been published^5^. This fetus, in which the presence of ZIKV was confirmed in brain tissue, displayed microcephaly, agyria, hydrocephalus, dystrophic calcifications in the cortex and subcortical white matter, focal inflammation and Wallerian degeneration of the descending corticospinal tract.

To meet the urgent need for models to investigate the link between ZIKV infection and abnormal brain development, several *in vitro* and *in vivo* models have recently been developed. Common themes have emerged across models of ZIKV infection in cultured human neural progenitor cells and brain organoids, as well as in immunocompromised and immunocompetent mouse models: efficient infection and replication of ZIKV in neural progenitor cells (NPCs)^8^,^9^,^10^,^11^,^12^,^13^, dysregulated cell cycle progression and growth in NPCs^8^,^9^,^10^,^11^,^12^,^13^, inhibition of NPC differentiation^9^, apoptosis in both NPCs and differentiated neurons^7^,^8^,^9^,^10^,^11^,^13^, elevated markers of neuroinflammation^14^,^15^, dysregulation of genes associated with cell cycle regulation, immune signaling, apoptosis and microcephaly^9^,^11^,^12^,^16^ and neurological symptoms in mice^14^,^15^,^17^,^18^. NPCs, brain capillaries and microglia have been shown to express high levels of candidate viral entry receptor AXL, suggesting a route for ZIKV infection in the developing brain^19^. Together, these studies paint a picture of catastrophic effects of ZIKV infection on brain growth, cell survival and inflammation and help establish a causal link between ZIKV and neurological conditions, including microcephaly.

Here we make use of a complementary animal model of ZIKV infection—intracranial injection of ZIKV in wild type C57BL/6 mice—to test the hypothesis that neurodevelopmental stages when the brain is undergoing rapid growth are particularly vulnerable to the effects of ZIKV infection. The peak of the brain growth spurt in rodents occurs during early postnatal life, with adult brain mass being largely attained by weaning at three weeks of age^20^,^21^,^22^. Thus, we infected mice with ZIKV at either postnatal day 7 (P7) or P21 and collected brain tissue for histological analysis 4 days post-inoculation. Although both time points displayed widespread immunoreactivity for ZIKV and the apoptosis marker cleaved caspse 3 throughout the brain, younger animals showed large increases in apoptosis in a subset of brain areas and cell types that we did not observe in older animals. Of particular relevance to postmortem findings in humans with ZIKV-associated microcephaly, we find that corticospinal pyramidal neurons are one such cell type. These results indicate that periods of rapid brain growth are especially susceptible to the neurodevelopmental effects of ZIKV infection and provide a useful model for exploring candidate mechanisms and therapeutics.

## Results and Discussion

### Intracranial injection of ZIKV during early postnatal life results in widespread infection of the C57BL/6 mouse brain

**Figure 1:**
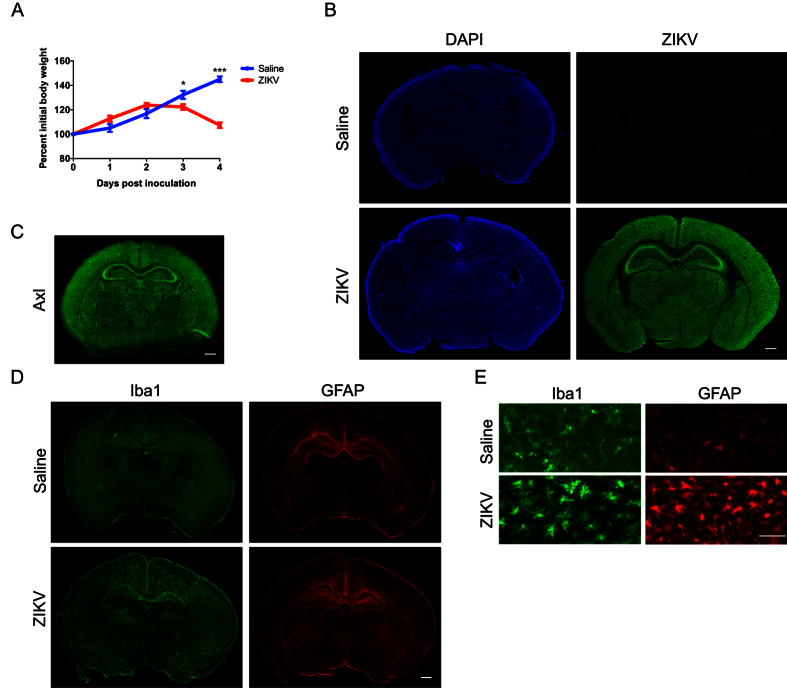
Intracranial injection of ZIKV results in widespread infection and activation of glia cells. Wild type C57BL/6 mice were injected intracranially with saline or ZIKV (MR766) at one week of age (postnatal day 7, P7), and brains were collected 4 days post-inoculation (dpi) for immunohistochemistry. (**A**) Graph showing percentage of initial body weight of saline or ZIKV injected mice at over 4dpi. Two-Way ANOVA revealed main effects on interaction, F(4,56) = 44.88, *p* < 0.001, time points, F(4,56) = 70.50, *p* < 0.001, treatments, F(1,14) = 7.42, *p* = 0.017. Sidak’s *post hoc* tests were used. *p < 0.05, and ***p < 0.001. N = 8 animals per group. (**B**) Images showing anti-ZIKV (green) and DAPI (blue) staining in saline and ZIKV injected wild type mouse brains. Scale bar: 500 μm. (**C**) Images showing anti-Axl (green) staining in a wild-type mouse brain at postnatal day 8. Scale bar: 500 μm. (**D**) Images showing anti-Iba1 (green) and anti-GFAP (red) staining in saline and ZIKV injected wild type mouse brains. Scale bar: 500 μm. (**E**) Higher magnification of anti-Iba1 (green) and anti-GFAP (red) staining in saline and ZIKV infected wild type mouse brains. Scale bar: 50 μm. Iba1-postive microglia exhibited morphology consistent with activation in ZIKV infected mice. Images are representative of 5-6 sections per animals from 6 animals per group (saline and ZIKV injected).

To test the effects of ZIKV infection at the peak of growth rate in the developing mouse brain, we performed intracranial injection of either saline or ZIKV (strain MR766) into postnatal day 7 (P7) wild type C57BL/6 mice. Infected mice stopped gaining weight from 2 days post-inoculation (2dpi) (Fig. 1A). By 4dpi, we observed severe loss of control of the body; infected animals had difficulty in remaining upright and exhibited circling movement, lying on their back (Movie 1). Control animals injected with saline were healthy throughout the experiment (Movie 2).

At 4dpi, we collected the brains of infected animals and controls for histological analysis. Immunostaining with anti-ZIKV antibody revealed evidence of widespread infection throughout the brain of ZIKV injected animals (Fig. 1B). Axl, a candidate entry receptor for ZIKV^19^, is distributed throughout the brain (Fig. 1C), consistent with widespread infection of ZIKV in the early postnatal mouse brain. In ZIKV infected animals we also observed a large increase in Iba1-positive microglia and glial fibrillary acidic protein (GFAP)-positive astrocytes (Fig. 1D) across the brain as compared to controls. Moreover, microglia in ZIKV infected brains appeared more active than in saline injected controls, as indicated by the morphology of Iba1-positve cells (Fig. 1E). Although it is possible some of the observed responses are due to the infusion of high virus inoculum, these results are consistent with a broad neuroinflammatory response to ZIKV infection.

### The brains of ZIKV infected mice are microcephalic and display non-uniform patterns of cleaved caspase 3 immunoreactivity

**Figure 2:**
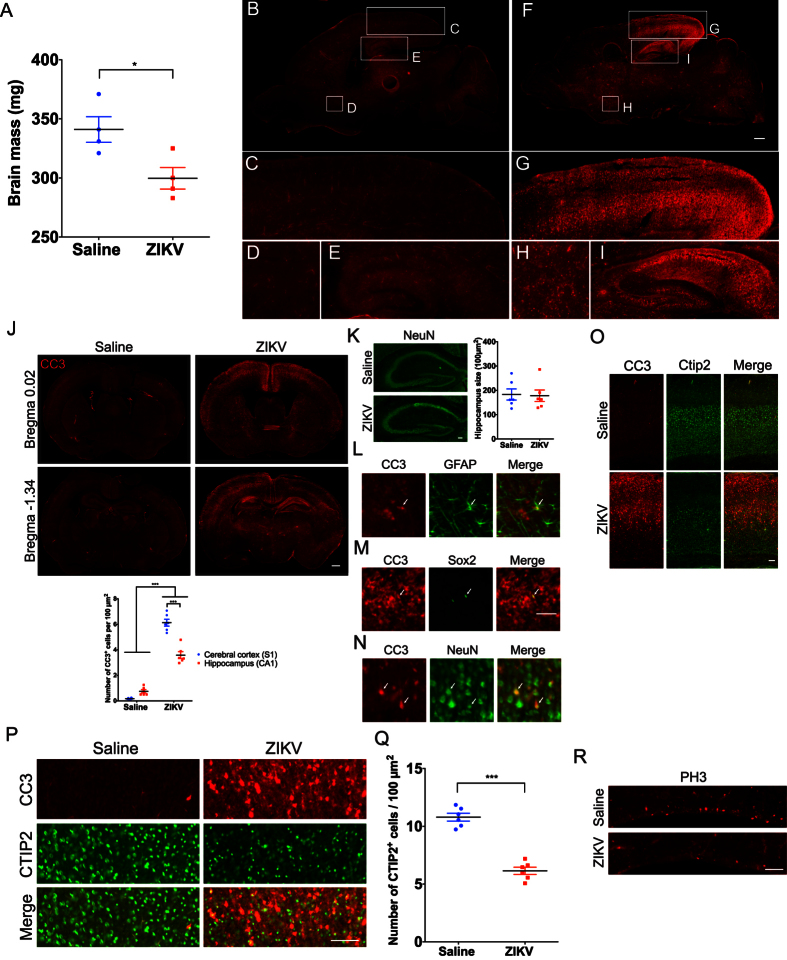
ZIKV infection results in decreased brain mass, non-uniform apoptosis, decreased CTIP2^+^ corticospinal neurons and depletion of proliferating cells in the ventricular zone. Brains of mice injected intracranially with saline or ZIKV at P7 and collected 4dpi for immunohistochemistry. (**A**) Graph showing reduced brain mass in animals injected with ZIKV at P7 as compared to saline injected controls. Independent sample t-tests were used. *p < 0.05. N = 4 animals per group. (**B,F**) Sagittal images of saline (**B**) and ZIKV (**F**) injected wild type mouse brains immunostained with anti-cleaved caspase 3 (CC3). Scale bar: 500 μm. Lettered boxes (C-E and G-I) in these images are magnified in corresponding panels below: occipital cortex (**C,G**), striatum (**D,H**), and hippocampus (**E,I**). ZIKV infected mouse brain showed widespread apoptosis, with occipital cortex and hippocampus showing particularly high levels of CC3^+^ cells. (**J**) Coronal images of saline and ZIKV injected wild type mouse brains stained with anti-CC3. Scale bar: 500 μm. CC3^+^ cells were enriched in upper layer II and layer V of the cerebral cortex, and CA1 regions of the hippocampus. Quantification of CC3^+^ cells in the primary somatosensory cortex (S1) and CA1 regions of the hippocampus. Two-way ANOVA and Tukey’s *post hoc* tests were used. F(1,20) = 59.58, *p* < 0.001 (interaction), F(1,20) = 470.4, *p* < 0.001 (treatment), and F(1,20) = 23.45, *p* < 0.001 (brain regions). ***p < 0.001. N = 6 animals per group. (**K**) Images showing NeuN staining (green) in the hippocampus of saline and ZIKV infected mouse brains. The size of the hippocampus is similar between saline and ZIKV injected mice. Independent sample t-tests were used. N = 6 animals per group. Scale bar: 100 μm. (**L–N**) Double immunostaining of anti-CC3 (red) with anti-GFAP (astrocyte marker, green) (**L**), anti-Sox2 (neural progenitor cell marker, green) (**M**), and anti-NeuN (neuronal marker, green) (**N**) in ZIKV infected mouse brains. Arrows indicate double labeled cells. Scale bar: 50 μm. (**O**) Images showing anti-CC3 (red) and anti-CTIP2 (green) staining in the motor cortex of saline and ZIKV injected wild type mice. Scale bar: 100 μm. (**P**) Images showing anti-CC3 (red) and anti-CTIP2 (green) staining in layer V of the motor cortex in saline and ZIKV injected wild type mice. Scale bar: 100 μm. Some, but not all, CC3^+^ cells were co-labeled with anti-CTIP2, a marker for subcortical-projecting neurons in the layer V cortex. ZIKV infected mice displayed reduced density of CTIP2^+^ neurons. Images are representative of 5-6 sections per animals from 6 animals per group (saline and ZIKV). (**Q**) Quantification of CTIP2^+^ cells in saline or ZIKV injected mouse brains. Independent sample t-tests were used. ***p < 0.001. N = 6 animals per group. (**R**) Images showing anti-phospho-histone H3 (PH3) staining in the ventricular zone of saline and ZIKV injected wild type mice. ZIKV infected mice displayed a reduced number of PH3^+^ cells. Scale bar: 100 μm.

Given the evidence linking ZIKV infection to microcephaly, we measured the mass of brains harvested from ZIKV infected mice at P7, 4dpi. We found that after only four days post-injection, brain mass was significantly decreased in ZIKV injected mice as compared to controls (Fig. 2A), demonstrating that ZIKV infection at P7 leads to microcephaly by 4dpi in wild type C57BL/6 mice.

As an initial exploration of mechanisms that may contribute to microcephaly in this model, we performed immunohistochemistry for cleaved caspase 3 (CC3), a marker of apoptosis. We found evidence of widespread apoptosis, as indicated by anti-CC3 staining, throughout the brain, with some brain regions having particularly high density of CC3^+^ cells, including the occipital cortex and hippocampus (Fig. 2B–I). Quantification of CC3^+^ cells in the primary somatosensory cortex and CA1 region of the hippocampus revealed that the density of CC3^+^ cells is greater in the cerebral cortex (Fig. 2J). Coronal sections of the infected mouse brains revealed a striking distribution of CC3^+^ cells in the cerebral cortex, with upper layer II and layer V containing a significantly higher density of CC3^+^ cells as compared to other cortical layers (Fig. 2J). This indicates that a variety of brain regions and cell types are vulnerable to apoptosis induced by ZIKV infection during early postnatal development.

A previous study using intracranial injection of ZIKV in newborn mice found hippocampal degeneration at 7dpi^23^. Although we did not observe a significant decrease in hippocampus size in ZIKV infected mice (Fig. 2K), the widespread apoptosis in the hippocampus suggests that this structure is undergoing degeneration at 4dpi.

To determine the cell types undergoing apoptosis after ZIKV infection, we double immunostained using anti-CC3 with anti-GFAP, which labels astrocytes, anti-NeuN, which labels neurons, or anti-Sox2, which labels neural progenitor cells. We only rarely observed GFAP^+^ or Sox2^+^ cells that were co-labeled with CC3 (Fig. 2L,M). In contrast, we observed widespread co-labeling of NeuN^+^ cells with CC3 (Fig. 2N). Thus, apoptosis occurs in some astrocytes and neural progenitor cells upon ZIKV infection, but to a lesser extent than in NeuN^+^ cells. These results indicate that that ZIKV infection in P7 mice leads to large-scale cell death in mature neurons.

Cortical layer V contains the primary output neurons in the cerebral cortex, including those that project to the spinal cord. Given the case report of Wallerian degeneration of the descending corticospinal tract in a ZIKV infected fetus^5^, we performed immunohistochemistry for CC3 and CTIP2, a marker for subcortical-projecting neurons, including corticospinal motor neurons^24^. We found that some, but not all, CC3^+^ cells were also CTIP2^+^ in the ZIKV infected mouse brains (Fig. 2O,P), indicating that corticospinal projection neurons are among the cell types undergoing apoptosis at this time point. Moreover, the density of CTIP2^+^ neurons is significantly decreased in the ZIKV infected mouse brains as compared to controls (Fig. 2O–Q), indicating a significant reduction in the number of corticospinal projecting neurons. This result is consistent with loss of bodily control in ZIKV infected mice (Movie 1). This observation has clear relevance to a prior report of corticospinal tract pathology in a fetus with microcephaly associated with ZIKV infection^5^.

One consideration in interpreting this result is that our analysis allows us to see a snapshot of cells undergoing apoptosis 4dpi in a mouse brain infected with ZIKV at P7. It is possible that the distribution of CC3 may well be different in other brain areas and cell types at other time points of infection and analysis. Indeed, this may be predicted based on results from other mouse models, which indicate that a broad variety of cell types in the brain are vulnerable to cell death induced by ZIKV infection^7^,^8^,^9^,^10^,^11^,^13^.

To understand whether ZIKV infection alters proliferation of progenitor cells in the postnatal ventricular zone (VZ) stem cell compartment^25^, we examined phosphorylated histone H3 (PH3), a mitotic marker. We found that PH3^+^ cells in the VZ are substantially reduced in ZIKV infected mice (Fig. 2R). This result indicates depletion of progenitor cells in early postnatal ZIKV infected mice. This is consistent with reports of cell cycle dysregulation in mouse models of ZIKV infection during early development^8^,^9^,^10^,^11^,^12^,^13^.

### Partial overlap between cleaved caspase 3 and ZIKV immunolabeled brain cells

**Figure 3:**
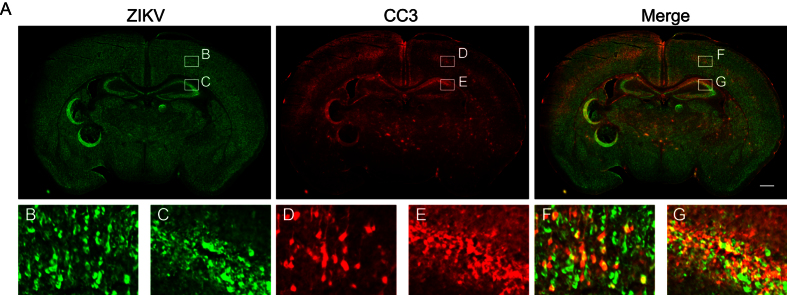
Partial overlap between cleaved caspase 3 and ZIKV immunolabeled brain cells. Brains of mice injected intracranially with saline or ZIKV at P7 and collected 4dpi for immunohistochemistry. Anti-CC3 staining is shown in red, and anti-ZIKV is shown in green. (**A**) Coronal image of a ZIKV infected mouse brain. Scale bar: 500 μm. Lettered boxes (**B–G**) in these images are magnified in corresponding panels below: cortex (**B,D,F**), and hippocampus (**C,E,G**). Some, but not all, CC3^+^ cells were positive for ZIKV. Images are representative of 5-6 sections per animals from 6 animals.

To test whether cells undergoing apoptosis in the developing brain are infected with ZIKV, we performed immunohistochemistry for ZIKV and CC3. We found that some, but not all, CC3^+^ cells are positive for ZIKV (Fig. 3A–G). One possible explanation for this incomplete overlap of CC3 and ZIKV is reduced ZIKV immunoreactivity in cells that are actively undergoing CC3-dependent (i.e. later stage) apoptosis, for example due to degradation of viral proteins. Another, not mutually exclusive, possibility is that ZIKV infection may be capable of inducing apoptosis through a non-cell autonomous mechanism, for example through bystander killing of uninfected cells.

### Intracranial injection of ZIKV in three week old mice results in a less severe induction of apoptosis than in one week old mice

**Figure 4:**
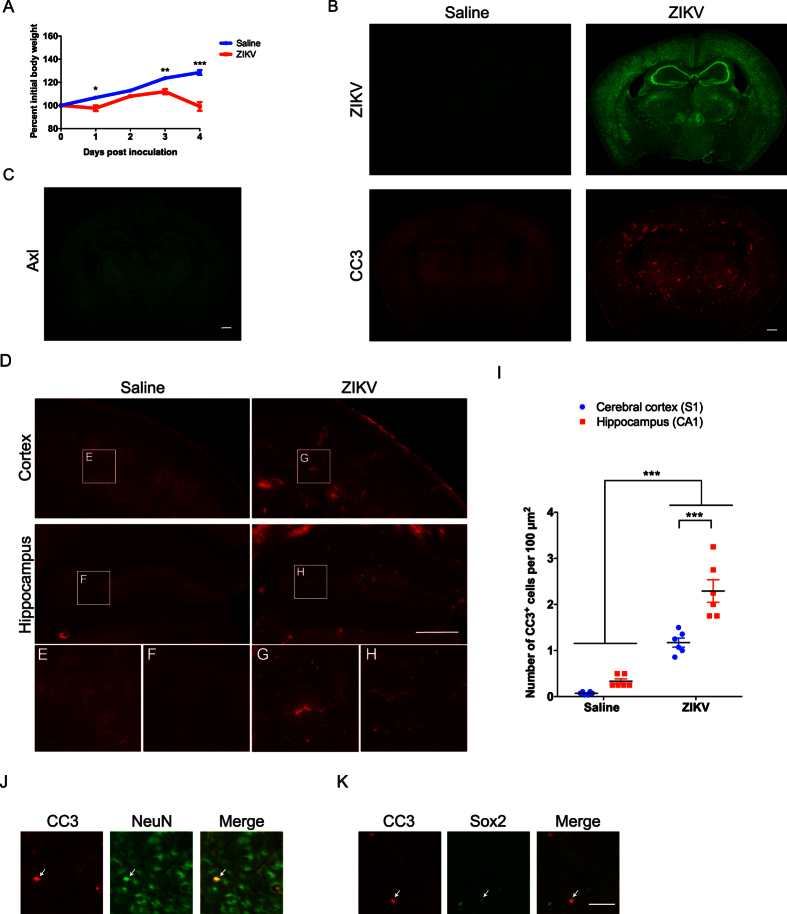
ZIKV infection in three-week-old mice results in a relatively uniform pattern of apoptosis. Wild type C57BL/6 mice were injected intracranially with saline or ZIKV at three weeks of age (P21), and brains were collected 4dpi for immunohistochemistry. (**A**) Graph showing percent initial body weight of saline or ZIKV infected mice over 4dpi. Two-Way ANOVA revealed main effects on interaction, F(4, 16) = 38.84, *p* < 0.001, time points, F(4, 16) = 71.56, *p* < 0.001, treatments, F(1, 4) = 28.02, *p* < 0.01. Sidak’s *post hoc* tests were used. *p < 0.05, **p < 0.01, and ***p < 0.001. N = 3 mice per group. (**B**) Coronal images of saline or ZIKV injected mouse brain. Anti-ZIKV staining is shown in green, and anti-CC3 is shown in red. Scale bar: 500 μm. (**C**) Images showing anti-Axl (green) immunostaining in a wild-type mouse brain at postnatal day 21. Scale bar: 500 μm. (**D**) Anti-CC3 staining in the cerebral cortex and hippocampus. Scale bar: 500 μm. Lettered boxes (**E–H**) in these images are magnified in corresponding panels below. ZIKV injected mouse brain exhibited increased CC3^+^ cells compared to saline injected ones, but the degree of apoptosis was less severe than that of one week old ZIKV injected mice. Images are representative of 4-5 sections per animals from 2 animals. (**I**) Quantification of CC3^+^ cells in the primary somatosensory cortex (S1) and CA1 regions of the hippocampus. Two-way ANOVA and Tukey’s *post hoc* tests were used. F(1, 20) = 10.12, *p* < 0.01 (interaction), F(1, 20) = 129.0, *p* < 0.001 (treatment), and F(1, 20) = 26.28, *p* < 0.001 (brain regions). ***p < 0.001. N = 6 brain sections from 3 animals per group. (**J,K**) Double immunostaining of anti-CC3 (red) with anti-NeuN (neuronal marker, green) (**J**), and anti-Sox2 (neural progenitor cell marker, green) (**K**) in ZIKV infected mouse brains. Arrows indicate double staining cells. Scale bar: 50 μm.

Finally, we asked whether ZIKV infection at three weeks of age (P21), a time when the mouse brain has largely attained adult size, has similar or different effects on apoptosis in the brain as compared to ZIKV infection at P7. We performed intracranial injection of either ZIKV or saline in P21 wild type C57BL/6 mice. ZIKV infected mice stopped gaining weight from 3dpi (Fig. 4A). By 4dpi, infected mice exhibited ruffled fur, kyphosis and severe paresis. Similar results were obtained for mice injected at two weeks of age (P14) (data not shown). Of note, these symptoms were overall less severe than those we observed in mice injected with ZIKV at P7. Control animals injected with saline were healthy throughout the experiment.

At 4dpi, we collected the brains of infected animals and controls for histological analysis. ZIKV immunoreactivity was broadly distributed throughout the cerebral cortex and hippocampus (Fig. 4B), although some subcortical areas appeared to have less signal as compared to mice infected with ZIKV at P7 (compare Figs 1B and 4B). To understand if the difference in ZIKV infection efficiency between P7 and P21 may be related to the expression pattern of Axl, we performed immunohistochemistry for Axl on P21 mouse brain. We found that Axl immunoreactivity is weak through out the brain at P21, indicating a relatively low level of Axl expression (Fig. 4C). Thus, it is possible that the difference on ZIKV infection efficiency in the subcortical brain regions between P7 and P21 may be due to levels of Axl expression.

Interestingly, the pattern of apoptosis in P21 infected brains was very different from that of P7 infected brains. We observed an increase in CC3^+^ cells throughout the brain as compared to controls (Fig. 4B); however, we did not observe the large increase in CC3^+^ cells in the upper layer II and layer V of the cerebral cortex and hippocampus that we observed in animals infected at P7 (Fig. 2). Overall, the magnitude of brain apoptosis in P21 ZIKV infected mice is less than what we observed in P7 ZIKV infected mice. Within the P21 brain, the density of CC3^+^ cells is higher in the hippocampus than in the cerebral cortex (Fig. 4D–I). In addition, we observed widespread labeling of NeuN^+^ cells with CC3, and only rarely observed Sox2^+^ cells that were co-localized with CC3 (Fig. 4J,K), indicating that ZIKV infection in P21 mouse brain also primarily leads to cell death in mature neurons. Given that P7 encompasses the peak of growth spurt in rodent brain, and that P21 is after the growth spurt completed, we interpret this as indicating that periods of high growth rate may be particularly vulnerable to neurological effects of ZIKV infection. This is consistent with clinical reports of more severe neurological effects of ZIKV infection in fetuses and newborns than in adults.

### Summary

In summary, we have reported the neurodevelopmental effects of intracranial injection of ZIKV in wild type C57BL/6 mice, a strain commonly used for neurobiological applications. Consistent with our initial hypothesis, there is a more severe apoptotic response to ZIKV infection at P7, when the brain growth rate is maximal, as compared to P21, when the brain has largely attained adult size. While ZIKV infection at P7 causes altered proliferation in cortical stem cells similar to what has been reported in other models, we also find that neurons that are post-mitotic but still actively increasing in size (e.g. CTIP2^+^ corticospinal pyramidal neurons) are susceptible to ZIKV-induced apoptosis. This extends our knowledge of cell types that are vulnerable to the effects of ZIKV infection to include not only proliferating neural progenitor cells, but also post-mitotic neurons that are undergoing rapid increases in cell size. This P7 intracranial infection model recapitulates aspects of human neuropathology associated with ZIKV infection, including degeneration of corticospinal neurons, and provides relatively easy to score phenotypes to study neurodevelopmental mechanisms of ZIKV infection. Furthermore, the robust apoptosis phenotype present in these animals provides a useful model for testing candidate therapeutics and for investigating whether some genetic backgrounds may confer enhanced susceptibility or resilience to the neurodevelopmental effects of ZIKV infection.

## Materials and Methods

### Cells and Virus stocks

ZIKV (MR766 strain, isolated from the blood of a sentinel rhesus monkey, Uganda, 1947) was obtained from World Reference Center for Emerging Viruses and Arboviruses at University of Texas Medical Branch. Virus was grown in Vero 76 cells (ATCC # CRL 1587) maintained in high glucose DMEM with 10% fetal bovine serum (FBS). The infectious virus titers were determined by plaque assays on Vero cells (ATCC # CCL 81) incubated for 4 days with 1% low melting point agarose overlay.

### Pathogenicity studies in mice

The timed pregnant C57Bl/6 females were purchased from Charles River Laboratories (Wilmington, MA) and maintained at the Animal Resources Center, TSRI Florida. One week old and three week old mice were intracranially (i.c.) inoculated, using 0.3 ml 31G syringes (one week old mice), and 1 ml 27½ G (three week old mice), with 30 μl of ZIKV (MR766, 1.9 × 10^7^ pfu/ml) or with saline following hypodermic anesthesia (one week old mice) and isoflurane anestheisa (three week old mice). Animals were observed daily for clinical illness. Necropsy of both the ZIKV infected and control animals were carried out at 4 day post inoculation (dpi). Mice injected at one week were given hydrothermic anesthesia 4dpi, and mice injected at three weeks were anesthetized with isoflurane and bled via cardiac puncture, followed by perfusion with ice cold phosphate bufferd saline (PBS) and 4% paraformaldehyde (PFA) at 4dpi. The brains were collected and placed in ice-cold PBS followed by overnight fixation in ice cold 4% PFA in PBS.

All research was conducted in accordance with National Institutes of Health and Association for Assessment and Accreditation of Laboratory Animal Care guidelines and approved by The Scripps Research Institute’s Institutional Animal Care and Use Committee.

### Immunohistochemistry

Immunostaining was performed as previously described^26^. Briefly, mouse brains were fixed in 4% paraformaldehyde for overnight, incubated in a 20% sucrose/PBS solution at 4 °C for three days, and embedded in Tissue-Tek OCT compound (Sakura). Coronal or sagittal sections (50 μm) were collected on Superfrost/Plus slides and immunostained using the following antibodies. Primary antibodies used in this study were anti-Zika (MR766) hyperimmune ascites (1:2000, World Reference Center for Emerging Viruses and Arboviruses), anti-Iba1 (1:500, Wako, 019-19741), anti-GFAP (1:2000, Life Technologies, 130300), anti-cleaved caspase 3 (1:2000, Cell Signaling Technology, 9661S), anti-CTIP2 (1:2000, Abcam, ab18465), anti-NeuN (1:1000, Millipore, MAB377), and anti-Sox2 (1:1000, Abcam, ab79351). Alexa Fluor 488, 594, and 647 conjugated secondary antibodies (Life Technologies) were used in this study. Immunofluorescent brain sections were counterstained with ProLong® Gold Antifade Mountant with DAPI (Thermo Fisher Scientific, P36931). Images were obtained with the Olympus VS120 microscope and processed using the VS-DESKTOP software (Olympus).

### Brain mass measurement

Mouse brains were fixed in 4% paraformaldehyde for overnight, incubated in a 20% sucrose/PBS solution at 4 °C for three days, and then brain mass was measured using a standard laboratory balance.

### Quantification of CTIP2^+^ neuronal density

Brain sections were immunostained with anti-CTIP2 antibody (1:2000 dilution). Regions of interest (ROIs) were cropped from primary somatosensory cortex barrel field (coronal section) using Photoshop (Adobe), and then the layer V cortex, as indicated by high levels of CTIP2 staining, was further cropped for density analysis. CTIP2^+^ neurons were manually counted using ImageJ (NIH) by experimenter who was blind to the experimental condition. Three ROIs were measured in each animal.

### Quantification of CC3^+^ cells

Brain sections were immunostained with anti-cleaved caspase 3 (CC3) antibody (1:2000 dilution). For cerebral cortex, CC3^+^ cells were counted in a 200 μm wide radial column of the primary somatosensory cortex barrel field (coronal section). For hippocampus, CC3^+^ cells were counted in a 200 m^2^ of CA1 region in hippocampus. VS-DESKTOP software (Olympus) was used to quantify CC3^+^ cells.

### Statistical analysis

Planned comparisons between treatments (saline or ZIKV) were performed for all assays using independent sample t-tests. Two-way ANOVA and Sidak’s *post hoc* tests were used to assess the body weight between saline and ZIKV injected mice over 4 days post injections. Two-way ANOVA and Tukey’s *post hoc* tests were used to assess the density of CC3 positive cells between saline and ZIKV infected mice in different brain regions. In all cases, normality was assessed using KS normality tests to ensure appropriate statistical tests were used. All statistics were performed using Graph Pad Prism 6, with significance set at *p* < 0.05.

## Additional Information

**How to cite this article**: Huang, W.-C. *et al*. Zika virus infection during the period of maximal brain growth causes microcephaly and corticospinal neuron apoptosis in wild type mice. *Sci. Rep.* **6**, 34793; doi: 10.1038/srep34793 (2016).

## Supplementary Material

Supplementary Information

Supplementary Movie 1

Supplementary Movie 2
