# Supplementary material for: Zika virus infection during the period of maximal brain growth causes microcephaly and corticospinal neuron apoptosis in wild type mice
Source: Sci Rep. 2016 Oct 7;6:34793. doi: 10.1038/srep34793 (PMC5054421; doi:10.1038/srep34793)
Supplement: Supplementary Information [file srep34793-s1.pdf]

Zika virus infection during the period of maximal brain growth causes microcephaly and corticospinal neuron apoptosis in wild type mice

Wen-Chin Huang[1,2], Rachy Abraham[3], Byoung-Shik Shim[3], Hyeryun Choe[3,4] & Damon T. Page[1,2,4]
[1]*Department of Neuroscience, The Scripps Research Institute, Jupiter, Florida*
[2]*The Doctoral Program in Chemical and Biological Sciences at The Scripps Research Institute, Jupiter, Florida*
[3]*Department of Immunology and Microbial Science, The Scripps Research Institute, Jupiter, Florida*
[4]*Authors for correspondence: Hyeryun Choe (HChoe@scripps.edu) or Damon T. Page (paged@scripps.edu)*

Video 1: Wild type C57BL/6 mice intracranially injected with ZIKV at P7, 4dpi. Mice display severe neurological symptoms.

Video 2: Wild type C57BL/6 mice intracranially injected with sailne at P7, 4dpi. Mice appear normal and do not show neurological symptoms.
